# The role of hypothalamic endoplasmic reticulum stress in schizophrenia and antipsychotic-induced weight gain: A narrative review

**DOI:** 10.3389/fnins.2022.947295

**Published:** 2022-09-16

**Authors:** Ruqin Zhou, Meng He, Jun Fan, Ruoxi Li, Yufeng Zuo, Benben Li, Guanbin Gao, Taolei Sun

**Affiliations:** ^1^School of Chemistry, Chemical Engineering and Life Sciences, Wuhan University of Technology, Wuhan, China; ^2^School of Public Health, Tongji Medical College, Huazhong University of Science and Technology, Wuhan, China; ^3^State Key Laboratory of Advanced Technology for Materials Synthesis and Processing, Wuhan University of Technology, Wuhan, China

**Keywords:** antipsychotics, schizophrenia, ER stress, obesity, inflammation, astrocytes

## Abstract

Schizophrenia (SCZ) is a serious mental illness that affects 1% of people worldwide. SCZ is associated with a higher risk of developing metabolic disorders such as obesity. Antipsychotics are the main treatment for SCZ, but their side effects include significant weight gain/obesity. Despite extensive research, the underlying mechanisms by which SCZ and antipsychotic treatment induce weight gain/obesity remain unclear. Hypothalamic endoplasmic reticulum (ER) stress is one of the most important pathways that modulates inflammation, neuronal function, and energy balance. This review aimed to investigate the role of hypothalamic ER stress in SCZ and antipsychotic-induced weight gain/obesity. Preliminary evidence indicates that SCZ is associated with reduced dopamine D2 receptor (DRD2) signaling, which significantly regulates the ER stress pathway, suggesting the importance of ER stress in SCZ and its related metabolic disorders. Antipsychotics such as olanzapine activate ER stress in hypothalamic neurons. These effects may induce decreased proopiomelanocortin (POMC) processing, increased neuropeptide Y (NPY) and agouti-related protein (AgRP) expression, autophagy, and leptin and insulin resistance, resulting in hyperphagia, decreased energy expenditure, and central inflammation, thereby causing weight gain. By activating ER stress, antipsychotics such as olanzapine activate hypothalamic astrocytes and Toll-like receptor 4 signaling, thereby causing inflammation and weight gain/obesity. Moreover, evidence suggests that antipsychotic-induced ER stress may be related to their antagonistic effects on neurotransmitter receptors such as DRD2 and the histamine H1 receptor. Taken together, ER stress inhibitors could be a potential effective intervention against SCZ and antipsychotic-induced weight gain and inflammation.

## Introduction

Schizophrenia (SCZ) is a severe mental disorder that affects approximately 1% of people worldwide. This disease is characterized by positive symptoms (e.g., hallucinations, delusion, thought disturbances, and disorganized speech and behavior); negative symptoms (e.g., poverty of speech, anhedonia, and apathy); and cognitive impairment associated with memory, attention, and executive function (McCutcheon et al., 2020). These symptoms impair social and occupational functioning and increase the risk for suicide, substantially reducing the lifespan of patients with SCZ. A body of evidence has revealed that dysregulation of neurotransmission pathways such as dopaminergic, glutamatergic, gamma-aminobutyric acid (GABA)-ergic, serotonergic, opioid, and cholinergic plays an important role in the pathogenesis of SCZ (McCutcheon et al., 2020). However, the etiology of SCZ remains elusive and new therapeutic strategies are urgently needed. Additionally, clinical evidence has consistently demonstrated that patients with SCZ have a higher risk of developing metabolic disorders such as obesity, glucose metabolic disorders, dyslipidemia, and hyperinsulinemia (Harris et al., 2013). Numerous studies have demonstrated that these metabolic side effects are mainly caused by antipsychotic drug treatment. However, growing evidence has revealed that patients with SCZ have an intrinsic metabolic risk (Freyberg et al., 2017). Researchers have reported that drug-naïve patients or patients from the pre-medication era have metabolic disorders such as a higher body mass index (BMI), waist circumference, and low-density lipoprotein (LDL) levels; hyperinsulinemia; and abnormalities in insulin secretion (Harris et al., 2013; Freyberg et al., 2017). There have been similar findings in youth (12–17 years old) compared with controls. Drug-naïve patients experiencing their first psychotic episode have a larger waist circumference and LDL levels (Jensen et al., 2017). However, the mechanisms underlying these abnormalities are not clear. The hypothalamus is well known to control a variety of body functions such as hunger, energy expenditure, the sleep/wake cycle, stress response, and reproduction, among others. Research suggests that the hypothalamus also mediates cognitive performance and psychosocial health (Burdakov and Peleg-Raibstein, 2020). Accumulated evidence indicates that patients with SCZ have abnormalities in gross anatomic regions (hypothalamus, the third ventricle, and the hypothalamic subregions such as the paraventricular nucleus [PVN]) as well as changes at the cellular level (reduction of PVN neurons, peptides, and neurotransmitters) (Bernstein et al., 2021). A recent clinical study reported a reduction in hypothalamic dopamine D2/D3 receptor (DRD2/DRD3) availability (examined by ^18^F-fluorodeoxyglucose and ^18^F-fallypride positron emission tomography [PET] imaging) in unmedicated patients with SCZ (Mitelman et al., 2020), suggesting the involvement of hypothalamic DRD2/DRD3 signaling in SCZ. Reduced DRD2 signaling in the hypothalamus is known to increase food intake, body weight, and regulate glucose metabolism (Ikeda et al., 2020), suggesting that the reduced DRD2 signaling may also be related to SCZ-related metabolic disorders such as increased BMI and abnormalities in glucose metabolism. However, the mechanisms are currently incompletely understood.

Antipsychotics are the mainstay of treatment for SCZ. However, almost all antipsychotics are associated with varying degrees of weight gain and even obesity (Barton et al., 2020). Clinical studies have reported weight gain ranging from 0.09 to 12.4 kg in patients taking antipsychotics such as olanzapine, clozapine, quetiapine, ziprasidone, risperidone, and haloperidol for 6 weeks to 1 year (Nasrallah, 2008; Barton et al., 2020). The weight gain liability of antipsychotics is clozapine ≈ olanzapine > zotepine > quetiapine > risperidone > ziprasidone > aripiprazole (Nasrallah, 2008; Stogios et al., 2022). Olanzapine is one of the most obesogenic antipsychotics. Previous studies have shown that approximately 67–90% of patients taking olanzapine gain at least 3.3–12 kg of body weight after 8 weeks to 12 months of olanzapine treatment (Eder et al., 2001; Nasrallah, 2008). Antipsychotic-induced weight gain/obesity is an important risk factor for type II diabetes, cardiovascular disease, stroke, and patient noncompliance, and leads to decreased life expectancy and increased mortality in patients with SCZ.

Significant efforts have been made to uncover the underlying mechanisms of antipsychotic-induced weight gain/obesity. Several neurotransmitter receptors that regulate food intake and energy expenditure are involved in antipsychotic-induced weight gain including the histamine H1 receptor (H1R), the serotonin 2C receptor (5-HT2CR), DRD2, the α2 adrenergic receptor, the muscarinic M3 receptor (M3R), the cannabinoid type 1 receptor (CB1R), the GABA type A receptor, and the melanocortin 4 receptor (MC4R) (Nasrallah, 2008). It has been reported that olanzapine, clozapine, risperidone, or quetiapine could block hypothalamic H1R or 5-HT2CR, thereby activating adenosine 5′-monophosphate (AMP)-activated protein kinase (AMPK) signaling and increasing neuropeptide Y (NPY) expression, which in turn leads to increased food intake, weight gain, and glucose intolerance (Ikegami et al., 2013; He et al., 2014; Wan et al., 2020). Another study reported that acute olanzapine and clozapine treatment tends but does not significantly induce AMPK activation in the hypothalamus (Fernø et al., 2011). However, pharmacological studies have shown that H1R agonists such as 2-((3-trifluoromethyl)phenyl) histamine dimaleate (FMPH) cannot pass through the brain-blood barrier (BBB) (Malmberg-Aiello et al., 1998), which has limited the use of H1R agonists in reducing antipsychotic-induced weight gain. Activation of hypothalamic H1R by using betahistine, an H1R agonist/H3R antagonist, partially inhibits olanzapine-induced weight gain in patients (Poyurovsky et al., 2013) and rodents (Deng et al., 2012). Our recent study reported that gold nanoclusters (AuNCs) could eliminate olanzapine-induced food intake and obesity in rats partly *via* affecting H1R-AMPK signaling (He et al., 2022b). Moreover, a 5-HT2CR-specific agonist, lorcaserin, decreases risperidone- and olanzapine-induced overeating and weight gain in rats (Lord et al., 2017; Wan et al., 2020). Co-treatment with cevimeline, an M3R agonist, attenuates olanzapine-induced weight gain *via* M3R-AMPK signaling in female rats (Han et al., 2022). Olanzapine treatment also decreases CB1R expression in the dorsal vagal complex (DVC) and the hypothalamus (Weston-Green et al., 2012; Lazzari et al., 2017). Co-treatment with a neutral CB1R antagonist and an inverse agonist, rimonabant and NESS06SM, respectively, significantly reduces olanzapine-induced weight gain in rats (Lazzari et al., 2017).

Antipsychotic-induced weight gain/obesity is also associated with several neuropeptides or hormones that regulate energy balance and neuroendocrine function such as proopiomelanocortin (POMC), NPY, AgRP, insulin, leptin, and ghrelin (Ballon et al., 2014). Olanzapine, clozapine, and risperidone treatment in rodents has been associated with increased expression of NPY and AgRP or decreased expression of POMC in the hypothalamus (Kirk et al., 2006; Fernø et al., 2011; Lian et al., 2015). Infusing an MC4R agonist, setmelanotide, reduces hyperphagia in risperidone-fed mice (Li L. et al., 2021). In rats, olanzapine treatment upregulates the messenger RNA (mRNA) and protein expression of growth hormone secretagogue receptor 1a (GHS-R1a), a receptor of ghrelin in the hypothalamus (Zhang et al., 2014). Cerebroventricular injection of a GHS-R1a antagonist, D-Lys3-GHRP-6, suppresses olanzapine-induced hyperphagia in rats, suggesting the involvement of ghrelin signaling in antipsychotic-induced weight gain (Zhang et al., 2014). A recent study reported that olanzapine activates hypothalamic NPY-AMPK signaling by disrupting the GHSR-H1R interaction, and this effect contributes to olanzapine-induced weigh gain (Chen et al., 2020). Antipsychotic-induced weight gain is associated with glucose metabolism disorders (Zhang et al., 2017a). Olanzapine, clozapine, risperidone, and quetiapine treatment has been associated with weight gain and abnormal blood glucose and leptin levels in patients (Doane et al., 2022) and rodents (Cope et al., 2005; Kirk et al., 2009). Anti-diabetic drugs such as metformin partially reduce olanzapine- or clozapine-induced weight gain in patients (Ellul et al., 2018) and rodents (Hu et al., 2014). Interestingly, evidence suggests that gut microbiome alterations are largely associated with the pathophysiology of SCZ as well as antipsychotic-induced obesity (Singh et al., 2022). Indeed, obesogenic antipsychotic treatment causes gut microbiota imbalance in both rodents and humans (Singh et al., 2022). Co-administration of the prebiotic Bimuno galacto-oligosaccharides (B-GOS^®^) alleviates olanzapine-induced weight gain in rats (Kao et al., 2018). Despite significant progress on the underlying mechanisms of antipsychotic-induced weight gain, there is still a lack of effective drugs for the treatment of antipsychotic-induced obesity.

Endoplasmic reticulum (ER) stress refers to physiological or pathological states in which proteins are over unfolded or misfolded in the ER. Hypothalamic ER stress plays a critical role in mediating neuroinflammation and neuronal injury (Yi et al., 2019), as well as regulating food intake, energy expenditure, and body weight (Ramírez and Claret, 2015). Several studies have investigated the association between ER stress and SCZ and have reported that genotypes of ER stress-related genes, including X-box-binding protein 1 (XBP-1) 116C/G and 197C/G, are causative factors of SCZ (Chen et al., 2004; Kakiuchi et al., 2004). An ER stress inhibitor 4-phenylbutyric acid (4-PBA) has been suggested as an important therapy to treat SCZ-related manifestations (Patel et al., 2017). The alterations of ER stress in the hypothalamus of patients with SCZ are not clear. SCZ has been reported to be accompanied by reduced hypothalamic DRD2 signaling (Mitelman et al., 2020), which is largely implicated in ER stress (Song et al., 2017), suggesting that hypothalamic ER stress may be involved in SCZ pathology and its associated metabolic disturbances. Moreover, the most widely used but obesogenic antipsychotics such as olanzapine induce activation of hypothalamic ER stress in rodents (He et al., 2019). Inhibition of ER stress suppresses olanzapine-induced hyperphagia and weight gain (He et al., 2019). These findings suggest the importance of hypothalamic ER stress in obesity induced by antipsychotics such as olanzapine. This review systematically elucidates the association between hypothalamic ER stress in SCZ and antipsychotic-induced weight gain/obesity, discussing the possible underlying molecular mechanisms and providing insights into the search for targets that could alleviate SCZ and antipsychotic-induced obesity.

## The role of hypothalamic endoplasmic reticulum stress in body weight control

The hypothalamus is an indispensable “headquarters” for regulating energy homeostasis (Tran et al., 2022). It contains a number of nuclei such as the arcuate nucleus (ARC), the PVN, the ventromedial hypothalamus (VMH), the dorsomedial hypothalamus (DMH), and the lateral hypothalamus (LH) to respond to hormones and nutrients and to regulate food intake and energy expenditure. The ARC which contains two major populations of “first-order” neurons with opposing effects on energy homeostasis: POMC neurons that express the anorexigenic peptides POMC and cocaine- and amphetamine-regulated transcript, and NPY/AgRP neurons that express the orexigenic peptides NPY and AgRP (Vohra et al., 2022). NPY/AgRP and POMC neurons send projections to “second-order” neurons in other hypothalamic regions such as the VMH, PVN, and LH, regulating body weight and glucose homeostasis (Vohra et al., 2022). Leptin and insulin are two important hormones that inhibit food intake and body weight by acting on the hypothalamus. Insulin and leptin receptors are expressed on POMC and AgRP neurons (Shin et al., 2017; Üner et al., 2019). By binding to their receptors, insulin and leptin directly stimulate anorexigenic POMC neurons and inhibit orexigenic NPY/AgRP neurons in the ARC to suppress food intake and to increase energy expenditure. Treatment with leptin or insulin increases POMC expression but decreases the release of AgRP (Breen et al., 2005). Mechanically, leptin and insulin reduce food intake and stimulate energy expenditure partly through AMPK, phosphoinositide 3-kinase (PI3K), suppressor of cytokine signaling 3 (SOCS3), protein tyrosine phosphatase 1B (PTP1B), and mammalian target of rapamycin (mTOR) signaling in the hypothalamus (Thon et al., 2016), and these effects play an essential role in leptin- and insulin-derived AgRP and POMC expression. Furthermore, other neurons such as dopaminergic and histaminergic neurons in the hypothalamus play an important role in regulating food intake and weight gain (Panula et al., 2000; Yonemochi et al., 2019). Hypothalamic dopamine suppresses food intake and weight gain by acting on postsynaptic DRD2 (Meguid et al., 2000). Hypothalamic histamine inhibits food intake and weight gain by activating H1R in the ARC and PVN (Panula et al., 2000). It has been reported that leptin and insulin regulate food intake and body weight partly by communicating with the hypothalamic DRD2 (Kleinridders and Pothos, 2019) and H1R pathways (Yoshimatsu et al., 1999).

In addition to neurons, growing evidence indicates that hypothalamic glia cells such as the astrocytes and microglia have a critical role in body weight regulation. Hypothalamic astrocytes and microglia accumulate and are activated in response to high-fat diet (HFD) (Zhang et al., 2017b; Valdearcos et al., 2019). Pharmacological deletion of astrocytes and microglia or reducing their capability for activating inflammation in the hypothalamus reduces food intake and weight gain and enhances leptin signaling in HFD-fed rodents (Valdearcos et al., 2019; Varela et al., 2021). Reactive astrocytes and microglia activate inflammatory signals such as inhibitor of nuclear factor kappa-B kinase subunit beta (IKKβ)/nuclear factor κB (NF-κB) signaling and produce inflammatory cytokines during HFD feeding (Valdearcos et al., 2019; Sa et al., 2022). Moreover, hypothalamic astrocytes reduce ghrelin-induced food intake by affecting AgRP neurons (Yang L. et al., 2015). Deletion of angiopoietin-like 4 in astrocytes increases the excitability and insulin sensitivity of POMC neurons and alleviates HFD-induced weight gain in mice (Varela et al., 2021). These findings suggest that astrocytes may partly regulate food intake and body weight by interacting with POMC and AgRP neurons.

### Hypothalamic neuronal endoplasmic reticulum stress and weight gain

**FIGURE 1:**
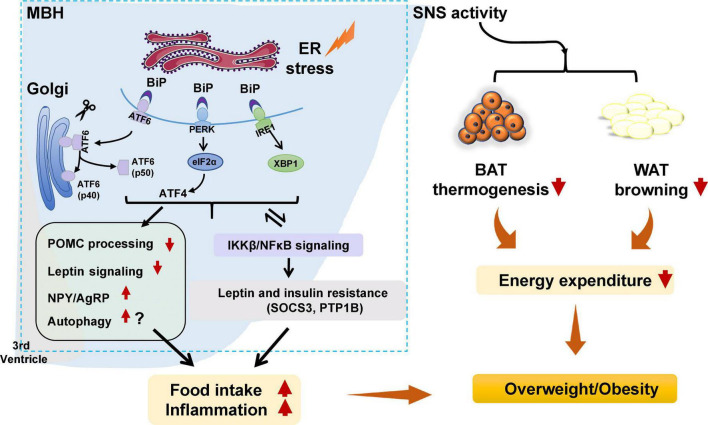
Potential role of hypothalamic endoplasmic reticulum (ER) stress in obesity and inflammation. Under certain stimuli, such as high-fat diet (HFD) feeding, drug treatment, or infection, ER stress is activated, and binding immunoglobulin protein (BiP) dissociates from protein kinase R-like ER kinase (PERK), inositol requiring enzyme 1 (IRE1), and activating transcription factor 6 (ATF6), resulting in the release of those three proteins. PERK is then activated by phosphorylation and p-PERK phosphorylates eukaryotic initiation factor 2α (eIF2α) and increases the translation of ATF4. ATF6 relocates to the Golgi apparatus and is processed by site 1 and 2 proteases, resulting in ATF6 activation. IRE1 is activated by phosphorylation. pIRE1 catalyzes X-box-binding protein 1 (XBP1) mRNA splicing, resulting in increased production of active spliced XBP1. These effects could (1) activate the hypothalamic autophagy signaling, impede proopiomelanocortin (POMC) processing, attenuate leptin signaling, and possibly increase neuropeptide Y (NPY), and agouti-related peptide (AgRP) expression, leading to increased food intake, decreased energy expenditure, inflammation, and weight gain; (2) decrease white adipose tissue (WAT) browning and brown adipose tissue (BAT) thermogenesis by affecting sympathetic nervous system (SNS) activity, resulting in lower energy expenditure and weight gain; and (3) trigger activation of the inhibitor of nuclear factor kappa-B kinase subunit beta (IKKβ)/nuclear factor κB (NF-κB) signaling pathway, resulting in hypothalamic leptin and insulin resistance (by affecting suppressor of cytokine signaling 3 [SOCS3] and protein tyrosine phosphatase 1B [PTP1B]). These effects could increase food intake, decrease energy expenditure, and promote inflammation, thus resulting in weight gain. Furthermore, activated IKKβ/NF-κB signaling could lead to ER stress in the hypothalamus and worsen hypothalamic inflammation.

Endoplasmic reticulum stress is an adaptive mechanism that refers to the pathophysiological process in which ER function is disturbed under conditions of hypoxia, oxidative stress, nutritional imbalance, or calcium homeostasis dysfunction (Schönthal, 2012). Mechanically, ER stress is mediated by protein kinase R-like ER kinase (PERK), activating transcription factor 6 (ATF6), and inositol requiring enzyme 1 (IRE1) (Cnop et al., 2012). Under unstressed conditions, these proteins bind to the chaperone BiP, also known GRP78 (Cnop et al., 2012). When ER stress is activated, GRP78 dissociates from PERK, IRE1, and ATF6. PERK is subsequently activated by phosphorylation. PERK phosphorylates the downstream eukaryotic initiation factor 2α (eIF2α) to increase the translation of activating transcription factor 4 (ATF4). Upon ER stress, ATF6 relocated to the Golgi apparatus and is processed by site 1 and 2 proteases; these changes form active ATF6 (p50), which is then transported to the nucleus to activate gene transcription (Matos et al., 2015). IRE1 could be activated by phosphorylation. Phospho-IRE1 cleaves 26 nucleotides from the mRNA of XBP1 and produces an active spliced form of XBP1 (Park et al., 2021). Then, these proteins activate comprehensive transcriptional and translational signaling, resulting in up- or downregulation of their downstream expression of signaling pathways (Kroeger et al., 2019; Figure 1).

Hypothalamic ER stress plays a crucial role in the development of obesity (Table 1). Mice with diet-induced obesity (DIO) have increased expression of hypothalamic ER stress markers, including phospho-PERK (p-PERK), phospho-eukaryotic initiation factor-2α (p-eIF2α), and phospho-IRE1 (p-IRE1) (Won et al., 2009; Cakir et al., 2013). Overnutrition activates hypothalamic PERK and promotes leptin resistance during obesity development in mice (Zhang et al., 2008). Pharmacologic activation of hypothalamic ER stress in rats results in hyperphagia and weight gain, and promotes leptin resistance (Ozcan et al., 2009). On the contrary, suppression of hypothalamic ER stress with ER stress inhibitors including tauroursodeoxycholic acid (TUDCA) or 4-PBA suppresses food intake in DIO mice (Ozcan et al., 2006). The mechanisms by which ER stress mediates feeding and body weight are not fully understood. Previous studies suggest that hypothalamic POMC neurons may be important response neurons. Diet-induced ER stress obstructs post-translational processing of POMC in mice (Cakir et al., 2013). Deletion of ATF4 from hypothalamic POMC neurons protects mice from obesity, glucose intolerance, and leptin resistance during HFD feeding (Xiao et al., 2017b). ATF4 also impairs hypothalamic α-melanocyte-stimulating hormone (α-MSH) production (Xiao et al., 2017a). Hypothalamic induction of ER stress attenuates POMC processing and decreases α-MSH levels by decreasing pro-converting enzyme 2 (Cakir et al., 2013). Hypothalamic ER stress could favor a positive energy balance by attenuating its response to an anorectic hormone leptin, whose receptors are expressed on POMC neurons (Cakir and Nillni, 2019). Activation of ER stress inhibits acute leptin signaling in arcuate POMC neurons (Williams et al., 2014). Constitutive expression of a dominant XBP1s form in POMC neurons protects mice from gaining weight by increasing energy expenditure and leptin sensitivity (Williams et al., 2014). Moreover, a recent study reported that genetic deletion of autophagy in POMC neurons of *ob*/*ob* mice worsens hyperphagia and reduces POMC neuronal projections to the PVN, characterized by less dense α-MSH-immunoreactive fibers (Park et al., 2020). The mechanisms by which autophagy reduces POMC neurons appear to involve inhibition of mTOR signaling, which is important for regulating cell growth and proliferation (Jaboin et al., 2007). Reduced POMC neuron projections would result in decreased expression of POMC and POMC-derived peptides such as α-MSH, leading to leptin resistance, hyperphagia, and weight gain (Oh et al., 2016). Neonatal TUDCA treatment ameliorates loss of autophagy-induced hyperphagia, weight gain, and the reduction in POMC neuronal projections is ameliorated (Park et al., 2020), suggesting that the ER stress-autophagy pathway in POMC neurons controls hypothalamic development and energy balance (Figure 1). Therefore, hypothalamic ER stress could mediate food intake and body weight by reducing POMC processing, activating autophagy, and inhibiting leptin signaling (Figure 1). Furthermore, ER stress could regulate food intake by affecting NPY/AgRP expression and neuronal function. NPY and AgRP are expressed in particular hypothalamic neurons that play an important role in feeding control (Morton and Schwartz, 2001). Induction of ER stress by tunicamycin significantly increases NPY and AgRP mRNA in the mouse hypothalamus (Ozcan et al., 2009). In AgRP neurons, RNA-seq revealed that ER-stress-related genes such as BiP and ATF6 are significantly activated during food deprivation (Henry et al., 2015). ATF4 knockout in AgRP neurons protects mice from weight gain by decreasing food intake and increasing energy expenditure (Deng et al., 2017). Additionally, in rodents fed a HFD, inhibition of ER stress reduces orexigenic NPY gene expression in the amygdala, suggesting a role for brain ER stress-NPY in regulating feeding (Areias and Prada, 2015). These findings suggest that ER stress could also regulate food intake *via* affecting AgRP and NPY.

**TABLE 1:** Findings highlighting role of hypothalamic ER stress-related proteins in mediating appetite and weight gain and their modifications by antipsychotic drugs.

References	Study design	ER stress-related markers	Physiological role	Alteration in schizophrenia	Alteration during antipsychotic treatment
Won et al., 2009	1. Mice were injected with leptin after pretreatment with tunicamycin in the third ventricle. 2. Mice fed a HFD were co-treated with 4-PBA. 3. WB and PCR were used to detect the expression of STAT3 related with leptin and insulin signaling and ER stress markers in the hypothalamus.	PERK ↑ eIF2α↑ IRE1 ↑ XBP-1 ↑ CHOP ↑ GRP78 ↑	ER stress induced central leptin and insulin resistance and increased food intake and weight gain.	NR	NR
Cakir et al., 2013	1. Rats fed a HFD were co-treated with TUDCA after central injection of leptin. 2. N43/5 cells were treated with tunicamycin or thapsigargin after pretreated with 4-PBA or salubrinal. 3. RT-qPCR, RIA, and WB were used to detect the expression of POMC-processing-related proteins and ER stress markers in the ARC and PVN of rats, and in N43/5 cells.	p-PERK ↑ p-eIF2α↑	ER stress obstructed the post-translational processing of POMC, and induced leptin resistance, therefore regulating feeding.	NR	NR
Ozcan et al., 2009	1. Mice with specific knockout of XBP1 in neurons fed with HFD and *ob/ob* mice pretreated with 4-PBA and leptin were used to examine the effect of XBP1 in leptin signaling. 2. GTT and ITT were used to detect glucose metabolism and insulin function. WB was used to analyze ER stress markers and LepRB and STAT3 in hypothalamus.	PERK ↑ IRE-1α↑ XBP1 ↑ CHOP ↑	Hypothalamic ER stress induced leptin resistance and impaired glucose homeostasis, resulting in weight gain.	NR	NR
Contreras et al., 2014	1. Rats were centrally injected with ceramide to induce ER stress. 2. Then, an adenovirus encoding GRP78 wild-type was injected into the VMH of these rats and obese Zucker rats with higher ceramide C16 and C18. 3. RT-PCR, WB and IHC were used to detect expression of ER stress markers in the MBH and VMH, UCP1 and FABP3 in BAT, and leptin and insulin signaling in the VMH.	GRP78 ↑ p-IRE1 ↑ p-PERK ↑ p-eIF2α↑ ATF6α↑ CHOP ↑	1. Hypothalamic ER stress decreased BAT thermogenesis. 2. GRP78 overexpression in the VMH improved leptin and insulin resistance, increased BAT thermogenesis, causing weight loss.	NR	NR
Contreras et al., 2017	1. Rats were fed a HFD to induce hypothalamic ER stress, then the rats were treated with TUDCA. 2. GRP78 adenovirus was injected into the VMH of rats. 3. GTT and ITT were used to detect insulin function and glucose metabolism. RT-PCR, WB, and IHC were used to detect expression of ER stress markers, BAT thermogenesis markers, and key proteins of leptin signaling in the VMH.	GRP78 ↑ p-IRE1 ↑ p-PERK ↑ p-eIF2α↑ ATF6α↑ CHOP ↑	1. Hypothalamic ER stress inhibited BAT thermogenesis and WAT browning, and induced leptin and insulin resistance. 2. GRP78 overexpression in the VMH improved leptin and insulin resistance, and increased BAT thermogenesis and WAT browning, causing weight loss.	NR	NR
Henry et al., 2015	1. AgRP and POMC neurons were dissociated from transgenic mice with food deprived. 2. RNA-seq was used to detect the expression of ER stress markers in AgRP and POMC neurons.	BiP ↑ IRE1 ↑ XBP1 ↑ ATF6 ↑	XBP1 in AgRP and POMC neurons regulated food intake.	NR	NR
Park et al., 2020	1. *Ob/ob* mice from P4 to P16 postnatal were treated with TUDCA and leptin. 2. *Ob/ob* mice exposed to insulin and glucose were treated with TUDCA. 3. *Ob/ob*-*POMC*-Cre-*ATG7*^loxP/loxP^ mice stimulated by glucose were treated with TUDCA. 4. GTT and ITT were used to detect insulin function and glucose metabolism. Using WB, RT-qPCR, IHC and M-FISH to examine ER stress markers and POMC projections in the hypothalamus.	ATF4 ↑ ATF6 ↑ GRP78 ↑ XBP1 ↑ CHOP ↑	ER stress inhibited leptin and insulin sensitivity, impaired glucose homeostasis and worsened POMC neuronal projections in the PVN, resulting in increased food intake and weight gain.	NR	NR
Deng et al., 2017	1. AgRP-ATF4 KO mice fed with HFD or injected with leptin were used to examine metabolic-related alteration. 2. AgRP-ATF4 KO mice were under cold exposure to detect thermogenic response. 3. GTT and ITT were used to detect insulin function and glucose metabolism. Using a rectal probe attached to a digital thermometer to measure rectal temperature in mice.	NR	AgRP ATF4 reduced insulin sensitivity, and decreased BAT thermogenesis and WAT browning.	NR	NR
Zhang et al., 2008	1. Normal chow-fed mice were injected with tunicamycin to induce ER stress. 2. Mice fed a HFD were injected with TUDCA. 3. IKKβ^CA^ was delivered bilaterally into the MBH of HFD mice, followed by insulin injection. 4. AgRP/IKKβ^lox/lox^ mice were fed with HFD. 5. Immunostaining and WB were used to examine the effects of IKKβ^CA^ on leptin and insulin signaling in the MBH. IP and WB were used to examine ER stress markers in the hypothalamus, and GTT was used to detect glucose metabolism.	p-PERK ↑ p-eIF2α↑	Hypothalamic ER stress activated IKKβ/NF-κB signaling, causing inflammation, glucose intolerance and central insulin and leptin resistance.	NR	NR
Wang et al., 2021	1. Rat primary astrocytes were pretreated with high glucose for 48 h, and then these cells were incubated with metformin for 1 h. 2. Rat primary astrocytes were treated with high glucose for 48 h after pretreatment with the AMPK activator AICAR for 1 h. 3. ELISA, co-IP, and WB were used to detect the ER stress markers and inflammatory cytokines in astrocytes.	p-PERK ↑ p-IRE1α↑ ATF6 ↑	Astrocytic ER stress induced inflammation by mediating AMPK.	NR	NR
He et al., 2021	1. Cultured astrocytes were treated with OLZ to detect the effects of OLZ on ER stress in astrocytes. 2. Rats were co-treated with OLZ and 4-PBA. 3. WB or IF were used to detect the expression of ER stress markers in astrocytes, and the GFAP, S100B, and TLR4 signaling in the hypothalamus.	p-PERK ↑ ATF6 ↑ IRE1 ↑ GRP78/BiP↑	1. ER stress induced astrocytes and TLR4 signaling activation. 2. Hypothalamic ER stress mediated food intake and body weight.	NR	1. OLZ induced astrocytic ER stress. 2. 4-PBA inhibited weight gain and astrocyte activation in the hypothalamus.
He et al., 2019	1. Rats were treated with OLZ for 1- and 8-day to detect the ER stress change in the hypothalamus. 2. OLZ and 4-PBA co-treated rats for 8 days. 3. WB was used to detect the expression of hypothalamic ER stress markers.	p-PERK↑ peIF2α↑ ATF4 ↑ GRP78/BiP ↑	Hypothalamic PERK-elF2α pathway mediated food intake and weight gain.	NR	OLZ activated hypothalamic PERK-eIF2α and IKKβ-NF-κB signaling.
Grajales et al., 2022	1. INS-1 cells were treated with OLZ or co-treated with OLZ and 250 μM TUDCA. 2. WB was used to detect the effect of OLZ in ER stress markers and ELISA was used to detect the concentration of insulin in INS-1 cells.	p-PERK ↑ p-eIF2α↑ IRE-1 ↑ XBP-1 ↑	ER stress inhibited insulin secretion.	NR	OLZ activated ER stress and inhibited insulin secretion, which were inhibited by TUDCA.
Lauressergues et al., 2012	1. Antipsychotics including DXMS, HAL, CLO, OLZ, RIS and QUE treated human hepatocyte cells for 24 h. 2. Rats were injected with CLO and OLZ for 1- and 3-h. 3. Hepatic lipid-related gene expression in cells and liver of rats was quantified by RT-qPCR and WB.	ATF4 ↑ CHOP ↑	ER stress activated the SREBP-1 and SREBP-2 pathways related to hepatic lipid accumulation.	NR	CLO, OLZ, and HAL activated the PERK pathway.

AgRP, agouti-related peptide; AgRP-ATF4 KO, agouti-related peptide neuron–specific ATF4 knockout; AgRP/IKKβ^lox/lox^, AgRP neuron-specific knockout of IKKβ; ARC, arcuate nucleus; AICAR, 5-aminoimidazole-4-carboxamide1-β-D-ribofuranoside; AMPK, adenosine 5′-monophosphate (AMP)-activated protein kinase; ATF4, activating transcription factor 4; ATF6, activating transcription factor 6; ATG7, autophagy related gene 7; BAT, brown adipose tissue; BiP, binding immunoglobulin protein; CHOP, C/EBP homologous protein; CLO, clozapine; co-IP, co-immunoprecipitation; DXMS, dexamethasone; ELISA, enzyme-linked immunosorbent assay; ER stress, endoplasmic reticulum stress; FABP3, fatty acid binding protein 3; GFAP, glial fibrillary acidic protein; GTT, glucose tolerance test; HFD, high-fat diet; IF, immunofluorescence; IHC, immunohistochemistry; IKKβ, inhibitor of nuclear factor kappa-B kinase subunit beta; IKKβ^CA^, constitutively active IKKβ; IP, immunoprecipitation; IRE-1, inositol requiring enzyme 1; ITT, insulin tolerance test; LepRB, leptin receptor; LPS, lipopolysaccharide; MBH, mediobasal hypothalamus; M-FISH, multiplex fluorescence in situ hybridization; NF-κB, nuclear factor κB; Ob/ob-POMC-Cre-ATG7^loxP/loxP^, leptin and POMC-specific ATG7 knockout; OLZ, olanzapine; PAKO, POMC neuron–specific ATF4 knockout; 4-PBA, 4-phenylbutyric acid; PCR, polymerase chain reaction; PERK, protein kinase R-like ER kinase; p-eIF2α, phosphorylated-eukaryotic initiation factor-2α; POMC, proopiomelanocortin; PVN, paraventricular nucleus; RIA, radioimmunoassay; RIS, risperidone; RNA-seq, RNA-sequencing; RT-qPCR, real-time quantitative polymerase chain reaction; S100B, S100 calcium binding protein B; SREBP-1, sterol regulatory element binding protein-1; SREBP-2, sterol regulatory element binding protein-2; STAT3, signal transducer and activator of transcription 3; TLR4, Toll-like receptor 4; TUDCA, tauroursodeoxycholic acid; UCP1, uncoupling protein 1; VMH, ventromedial nucleus of the hypothalamus; WAT, white adipose tissue; WB, western blotting; XBP-1, X-box-binding protein 1; NR, not reported.

The role of ER stress in other hypothalamic neuronal systems involved in metabolism and body weight regulation have not been studied extensively. The available evidence suggests that ER stress is related to several neurotransmitter receptors that play essential roles in regulating food intake and body weight such as DRD2, H1R, and CB1R. One study reported that the DRD2 agonist (bromocriptine) decreases the expression of GRP78/BiP in cultured cells, indicating that ER stress could be inhibited by DRD2 activation (Henderson et al., 2021). A study also reported that H1R antagonism induces ER stress in cultured cells (Jakhar et al., 2016). The CB1R agonist arachidonyl-2′-chloroethylamide (ACEA) attenuates ER stress and inflammation in Neuro-2a neuroblastoma cells (Vrechi et al., 2018). These findings suggest that the ER stress could be the downstream effect of DRD2, H1R, and CB1R signaling. Future studies should investigate whether hypothalamic ER stress could be mediated by altering hypothalamic DRD2, H1R, and CB1R signaling and its potential role in food intake and body weight regulation.

Hypothalamic ER stress also drives obesity by reducing energy expenditure (Table 1). Pharmacological activation of ER stress in the hypothalamus by ceramides effectively reduces brown adipose tissue (BAT) thermogenesis, resulting in weight gain (Contreras et al., 2014). Genetic overexpression of GRP78 in the VMH reduces ceramide-induced ER stress and increases BAT thermogenesis, resulting in weight loss in rats (Contreras et al., 2014). In HFD-induced obese rats, overexpression of GRP78 in the VMH attenuates ER stress, increases BAT thermogenesis, and stimulates white adipose tissue (WAT) browning, ultimately attenuating HFD-induced obesity (Contreras et al., 2017). Moreover, the study revealed that ER stress in the VMH decreases sympathetic nervous system (SNS) activity to inhibit BAT function and increases WAT browning, and these effects could be independent of feeding and leptin (Contreras et al., 2017). Treatment with TUDCA, an ER stress inhibitor, reduces food intake and tends to increase oxygen consumption in DIO mice (Cakir et al., 2013). Intracerebroventricular (ICV) administration of TUDCA induces weight loss, decreases hypothalamic ER stress, and elevates BAT temperature in rats (González-García et al., 2018). Furthermore, hypothalamic POMC neuron-specific ATF4 knockout protects DIO mice from obesity by increasing BAT thermogenesis and increasing oxygen consumption (Xiao et al., 2017b). ATF4 knockout in AgRP neurons promotes mouse fat loss mainly by increasing energy expenditure (Deng et al., 2017). Induction of XBP1s in POMC neurons significantly increases the metabolic rate to mediate thermogenesis in both BAT and inguinal WAT (Williams et al., 2014), therefore protecting mice against DIO. These findings suggest that hypothalamic ER-stress-induced reductions in energy expenditure may be at least partially related to the hypothalamic POMC and AgRP (Figure 1).

In the pathogenesis of obesity, hypothalamic ER stress is significantly linked to inflammation. Activation of hypothalamic inflammation leads to central metabolic dysregulations, uncoupling of food intake and energy expenditure, and weight gain (Jais and Brüning, 2017; Le Thuc et al., 2017). ER stress markers including PERK, ATF6, and IRE1 participate in activating the inflammatory processes (Garg et al., 2012). In the progression of obesity, a HFD could induce hypothalamic ER stress, and this effect could promote hypothalamic inflammation in mice (Zhang et al., 2008) and rats (Ropelle et al., 2010). Activation of hypothalamic ER stress *via* overnutrition activates IKKβ-NF-κB signaling, whereas inhibition of ER stress *via* intraventricular injection of TUDCA suppresses hypothalamic IKKβ-NF-κB signaling, reduces food intake, and induces weight loss in DIO mice (Zhang et al., 2008). This study also revealed that activation of IKKβ-NF-κB signaling by ER stress induces leptin and insulin resistance by affecting SOCS3, and these effects cause energy imbalance and weight gain (Zhang et al., 2008). Interestingly, ER stress is also regulated by inflammation. A study revealed that administration of a small molecule inhibitor of NF-κB, withaferin A (WA), reduces the expression of ER stress hallmarkers including PERK, XBP1, and ATF6 in the mouse pancreas (Kanak et al., 2017). An intraperitoneal injection of active IKKβ (IKKβ^CA^) lentivirus increases phosphorylation of PERK and eIF2α (Zhang et al., 2008). In rats, a single intraperitoneal injection of tumor necrosis factor alpha (TNF-α) increases expression of p-PERK, IRE1α, and GRP78 in the hypothalamus (Denis et al., 2010). Palmitate and TNF-α treatment upregulate NF-κB expression and ER stress-related gene expression in hypothalamic appetite-stimulating NPY/AgRP neurons (Dalvi et al., 2017). Therefore, ER stress and NF-κB signaling positively regulate each other during HFD feeding, and these effects induce a positive energy balance and cause obesity (Zhang et al., 2008; Figure 1).

### Hypothalamic astrocytic endoplasmic reticulum stress and weight gain

**FIGURE 2:**
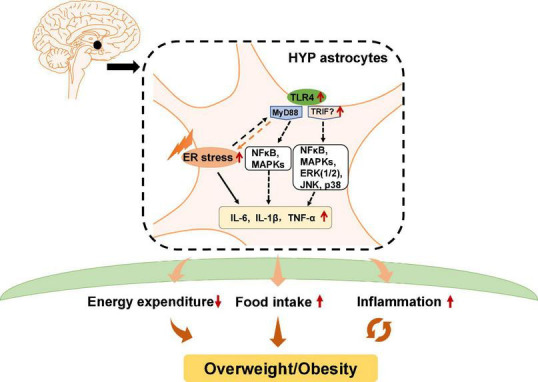
Potential role of astrocytic endoplasmic reticulum (ER) stress and Toll-like receptor 4 (TLR4) signaling in obesity and inflammation. In hypothalamic astrocytes, ER stress may activate TLR4 that then activates myeloid differentiation primary response 88 (MyD88)-independent and MyD88-dependent signaling, which increases the expression of nuclear factor κB (NF-κB), mitogen-activated protein kinases (MAPKs), extracellular signal-regulated kinase 1/2 (ERK1/2), c-Jun N-terminal kinase (JNK), and p38. These effects upregulate pro-inflammatory cytokines such as interleukin 6 (IL-6), IL-1β, and tumor necrosis factor alpha (TNF-α), and causing central inflammation. Activated astrocytic TLR4 signaling could also lead to decreased energy expenditure and promote ER stress activation, which increases food intake, eventually leading to obesity.

In addition to ER stress in hypothalamic neurons, ER stress in hypothalamic astrocytes has recently gained attention for its important role in the development of obesity and inflammation (Figure 2). Astrocytes are the most abundant glial cells in the central nervous system (CNS). Numerous studies have demonstrated the importance of hypothalamic astrocytes in regulating feeding (Kim J. G. et al., 2014), energy expenditure (Manaserh et al., 2020), and inflammation (Douglass et al., 2017). For example, fasting activates hypothalamic astrocytes (Chen et al., 2016). Astrocyte insulin receptor deletion in mice reduces energy expenditure and temperature (Manaserh et al., 2020). Researchers have reported that chronic and acute (single) HFD exposure in rodents induce inflammation and astrocytes activation in the hypothalamus (Zhang et al., 2017b; Cansell et al., 2021). Mice fed a HFD for 28 days had increased TNF-α mRNA expression in hypothalamic astrocytes (Sugiyama et al., 2020).

Previous studies have reported that in astrocytes, TLR4 signaling plays an essential role in obesity pathogenesis and inflammation (Figure 2). TLR4 is expressed by activated astrocytes (Shen et al., 2016). Growing evidence suggests that astrocytic TLR4 could be activated in response to natural ligands and existing compounds, such as tenascin C, damage-associated molecular patterns (DAMPS), lipopolysaccharide (LPS), and saturated fatty acids (SFA), which are well known to promote inflammation or induce weight gain. For example, tenascin C, an endogenous activator of TLR4, induces IL-6 expression in primary cortical astrocytes (Krasovska and Doering, 2018). LPS, which induces hypothalamic inflammation (Rorato et al., 2017), acts on the TLR4-myeloid differentiation factor 2 (MD2) complex (the binding site of LPS) to activate TLR4 and enhances IL-1β, IL-6, and TNF-α expression (Wu et al., 2012). DAMPs such as the high mobility group box protein 1 (HMGB-1) has been reported to activate TLR4 in mixed cultures of astrocytes and microglia, thereby inducing NF-κB activation (Rosciszewski et al., 2019). SFA, which is known to cause hypothalamic inflammation and obesity, activates TLR4 to induce an inflammatory response (Milanski et al., 2009). Moreover, a clinical study reported that obese patients have increased tenascin C and TLR4 levels in visceral adipose tissue (Catalán et al., 2012). LPS, total free fatty acid levels, and TLR4 mRNA are significantly increased in the plasma of patients with nonalcoholic steatohepatitis (Sharifnia et al., 2015). Serum from obese patients shows TLR4/NF-κB signaling activation in THP-1 monocytes (Yao et al., 2010). SFA induces pro-inflammatory cytokines secretion including TNF-α, IL-6, and IL-8 in human placental cells *via* activating TLR4 signaling (Yang X. et al., 2015). These findings suggest the importance of TLR4 in the pathogenesis of inflammation and obesity in humans. However, the role of hypothalamic astrocyte TLR4 signaling in inflammation and obesity is not clear in humans.

Toll-like receptor 4 has a cytoplasmic Toll/interleukin-1 receptor/resistance protein (TIR) domain and interacts with two adaptor molecules, namely myeloid differentiation primary response 88 (MyD88) (Medzhitov et al., 1998) and TIR domain-containing adapter protein-inducing interferon-β (TRIF) (Zuany-Amorim et al., 2002). In the MyD88-dependent pathway, TLR4 activation induces early recruitment of MyD88 and rapidly activates NF-κB and mitogen-activated protein kinases (MAPKs), therefore inducing inflammatory factors expression such as TNF-α and interleukin (IL)-1β, IL-6, and IL-15 (Gorina et al., 2011). In the MyD88-independent pathway, TLR4 interacts with TRIF and activates interferon regulatory factor 3 (IRF3), NF-κB, and MAPKs, including extracellular signal-regulated kinase 1/2 (ERK1/2), c-Jun N-terminal kinase (JNK), and p38, among others, thereby inducing the secretion of inflammatory factors such as TNF-α, IL-1β, and IL-6. Pharmacologic inhibition of TLR4, TLR4 knockout, TLR4-interactor knockdown, or hypothalamic ARC-restricted TLR4 knockdown reduces food intake, increases whole-body energy expenditure, reduces hypothalamic inflammation, and protects rodents from HFD-induced obesity (Milanski et al., 2009; Camandola and Mattson, 2017; Zhao et al., 2017; de Vicente et al., 2021). MyD88 deficiency in the mouse CNS (MyD88^ΔCNS^) or astrocyte-specific MyD88 knockout protects mice from chronic HFD-induced obesity, and mice exhibit ameliorated hypothalamic reactive gliosis and inflammation (Jin et al., 2020). *Trif* deficient (*Trif^–/–^*) mice show increased food intake and weight gain (Yang and Fukuchi, 2020). However, the role of hypothalamic TRIF signaling in food intake and weight gain is unclear. Moreover, activation of astrocytic IKKβ-NF-κB signaling increases fat mass and causes weight gain (Zhang et al., 2017b). Astrocytic IKKβ-NF-κB loss of function counteracts DIO in mice (Zhang et al., 2017b). Astrocyte-specific deletion of IKKβ in mice after 6 weeks of HFD feeding decreases hypothalamic inflammation and astrocytosis in the hypothalamic ARC, therefore reducing food intake and increased energy expenditure (Douglass et al., 2017). These findings suggest that astrocytes may regulate food intake, energy expenditure and inflammation development at least partly *via* TLR4 signaling, which involves the participation of MyD88, TRIF, and IKKβ-NF-κB signaling (Figure 2).

It is worth noting that TLR4 activation is associated with ER stress. TLR4 activation in HFD-induced obese mice could precede hypothalamic ER stress (Milanski et al., 2009). Inhibition of TLR4 signaling by anti-TLR4 antibodies (Milanski et al., 2009) or TLR4 knockout (Pierre et al., 2013) attenuates ER stress and reduces HFD-induced ER stress. Downstream molecules of TLR4 including MAPK and NF-κB, and the expression of inflammatory factors such as IL-1β and IL-6, could be activated by ER stress (Darling and Cook, 2014; Kim S. et al., 2014). Moreover, ER stress could mediate inflammation *via* TLR4 signaling during the development of obesity (Li, 2018). HFD-induced obese mice show significantly increased mRNA and protein expression of TLR4, TNF-α, and IL-6 in adipose tissue, while these changes are suppressed by treatment with the ER stress inhibitor 4-PBA (Li, 2018). TUDCA inhibits NF-κB activation in astrocytes induced by combined stimulation of LPS and interferon-γ (IFN-γ) (Yanguas-Casás et al., 2014), suggesting that inhibition of ER stress decreases astrocyte inflammation. These studies clarify the important relationship between ER stress and TLR4 signaling (Figure 2). In conclusion, hypothalamic ER stress in neurons and astrocytes has an important role in the regulation of body weight and inflammatory responses.

While it is known that ER stress induces inflammation in neurons and astrocytes, are these effects dependent or independent? Studies in cultured neurons have revealed that ER stress activates NF-κB signaling and increases expression of pro-inflammatory cytokines, and these effects could be inhibited by co-treatment with ER stress inhibitors such as 4-PBA (Wang et al., 2016; He et al., 2019). Studies in astrocytes have shown the same results: Activation of ER stress triggers an inflammatory response, which is inhibited by co-treatment with ER stress inhibitors (Yanguas-Casás et al., 2014; He et al., 2021). These findings suggest that ER-stress-induced inflammation in neurons and astrocytes could be independent. However, astrocytes and neurons are known to communicate with each other (Paixão and Klein, 2010). Research has proved that ER stress is transmissible between astrocytes and neurons (Sprenkle et al., 2019). ER stress in astrocytes/neurons could regulate inflammatory and ER stress in other astrocytes/neurons (Sprenkle et al., 2019). In astrocytes, ER stress activation augments inflammatory signaling and increases the expression and secretion of pro-inflammatory cytokines such as IL-6 and IL-1β (Wang et al., 2018). The receptors for IL-1β, and TNF-α are expressed on neurons (Friedman, 2001; Chadwick et al., 2008). It is possible that pro-inflammatory cytokines secreted by astrocytes can act on their receptors on neurons to stimulate neuronal ER stress, triggering neuronal inflammatory responses. It has been suggested that overnutrition/HFD activates astrocytes and microglia to release cytokines, and these cytokines then mediate inflammation in POMC and AgRP neurons and cause leptin and insulin resistance, resulting in impaired metabolism and weight gain (Ullah et al., 2021). Furthermore, the receptors for the above pro-inflammatory cytokines are also expressed on astrocytes (Friedman, 2001; Bobbo et al., 2021). Therefore, pro-inflammatory cytokines including IL-6, IL-1β, and TNF-α secreted by neurons may also activate ER stress in astrocytes to induce an inflammatory response. Taken together, during obesity development, hypothalamic inflammation could be a combined effects of ER stress activation in both neurons and astrocytes, and they could both trigger each other.

Besides astrocytes, other glia cells such as microglia, hypothalamic neural stem cells, and NG2 cells play a role in metabolic inflammation. Obesity activates microglia and inflammation in the hypothalamus (Mendes et al., 2018), whereas deletion of hypothalamic microglia abrogates inflammation in rodents (Valdearcos et al., 2014). NG2 glia cells, which express inflammatory cytokines such as IL-1β (Galichet et al., 2021), are increased in the brain of HFD-fed mice (Xiao et al., 2018). Neural stem cells, which express IL-6, IL-1β, and TNF-α (Chang and Kong, 2019), are reduced by HFD exposure *via* IKKβ/NF-κB signaling (Livesey, 2012). The role of ER stress in mediating inflammation in microglia, N2 glia, and neural stem cells has not been fully studied; and the available information is inconsistent. Increased ER stress in microglia contributes to neuroinflammation induced by paraquat (an herbicide) (Yang et al., 2020). Inhibition of ER stress *via* TUDCA reduces activation of microglial NF-κB induced by LPS and IFN-γ (Yanguas-Casás et al., 2014). However, LPS-induced increased IL-1β, IL-6, and TNF-α expression in microglia are partially reversed by tunicamycin (an ER stress inducer) (Wang et al., 2017). Inhibition of ER stress by 4-PBA promotes LPS-induced inflammation in cultured microglia (Wang et al., 2017). Furthermore, whether ER stress in hypothalamic microglia, N2 glia, and neural stem cells plays a role in metabolic inflammation and obesity remains to be uncovered. How antipsychotics affect ER stress in these glia cells and its potential role in antipsychotic-related inflammation is unknown. Astrocytes, which make up the largest glial population, are related to ER stress signaling in inflammatory regulation (Martin-Jiménez et al., 2017). Obesogenic antipsychotics such as olanzapine activate astrocytic ER stress, leading to weight gain (He et al., 2021). Therefore, the next section focuses on the role of astrocytic ER stress in antipsychotic-related inflammation and weight gain.

## The role of hypothalamic endoplasmic reticulum stress in schizophrenia and antipsychotic-induced weight gain

There are numerous studies reporting that SCZ is associated with metabolic disorders. Almost all antipsychotics cause weight gain/obesity, and this effect is the main cause of metabolic disorders in SCZ patients. Hypothalamic ER stress is well known in regulating food intake, energy expenditure, inflammation, and insulin and leptin signaling (Cakir and Nillni, 2019). In this section, we discuss the relationship between ER stress and SCZ and antipsychotic-induced weight gain.

### Insights from clinical studies on endoplasmic reticulum stress in schizophrenia and antipsychotic-induced weight gain

Several studies have investigated the relationship between ER stress and SCZ (Table 2). A study in B lymphocytes from patients with bipolar disorder reported that XBP1 and CHOP are upregulated upon treatment with ER stress inducers (thapsigargin and tunicamycin) (So et al., 2007), suggesting the ER stress could occur in patients with psychiatric disorders. XBP1 polymorphisms are associated with psychiatric disorders including SCZ, depression, and bipolar disorder in patients with or without antipsychotic treatment (Table 2). GRP78/BiP is an essential HSP70 resident protein in the ER. HSP70 is overexpressed in patients with SCZ and is suggested to be involved in the pathology of the disease (Table 2). Another study in a Korean population showed that HSP70-2 gene polymorphism might be related to the pathogenesis of SCZ (Table 2). Moreover, an autopsy study reported that in the dorsolateral prefrontal cortex of antipsychotic-treated SCZ patients, expression of GRP78/BiP and sXbp1/uXbp1 were increased (Table 2). These findings suggest that brain ER stress may play an important role in the pathophysiology of SCZ. However, in these studies certain subjects had been treated with antipsychotics; therefore, antipsychotics could have affected these results. Currently, there is a lack of direct clinical evidence regarding whether hypothalamic ER stress is related to the pathophysiology of SCZ. It has been reported that DRD2/DRD3 availability is significantly decreased in the hypothalamus of SCZ patients not receiving antipsychotic treatment (Mitelman et al., 2020). In mice lacking the dysbindin-1 gene, an animal model of SCZ, there are reduced dopamine levels in the hypothalamus (Hattori et al., 2008). These findings suggest that SCZ reduces DRD2/DRD3 signaling in the hypothalamus. Given that ER stress could work downstream of DRD2 (Song et al., 2017), it is possible that DRD2-ER stress pathway mediates SCZ. Moreover, it is currently unclear that how antipsychotics affect the hypothalamic ER stress in SCZ patients and whether it is involved in antipsychotic-induced obesity. The most widely used antipsychotics such as olanzapine, quetiapine and risperidone are known DRD2 antagonists. Therefore, it is possible that antipscyhotics may mediate the hypothalamic ER stress in SCZ patients. Furthermore, there is no clinical evidence of how hypothalamic astrocytes would be affected in patients with SCZ. The role of astrocytic ER stress in this population is currently not clear.

**TABLE 2:** Clinical studies of ER stress proteins in schizophrenia patients treated with or without antipsychotics.

References	Study location	Study design	ER stress-related protein/genes	Findings	Antipsychotic treatment
Kim et al., 2021	United States	UPR protein expression in the DLPFC of 22 matched pairs of elderly control subjects and subjects with SCZ was analyzed by WB and RT-qPCR.	sXBP1/uXBP1 ↑ GRP78/BiP ↑	1. In SCZ, BiP expression was increased, p-IRE1α expression was decreased, and PERK expression positively related to age was decreased. 2. There were decreased p-JNK2 and increased sXBP1, and IRE1α increased XBP1 mRNA splicing and drove upregulation of sXBP1 protein expression.	YES
Kakiuchi et al., 2004	Japan	The *XBP1*-116C/G polymorphism in 234 unrelated patients was genotyped by PCR, and Fisher’s exact test was used to examine the differences in genotype and allele frequencies.	*XBP1*-116C/C ↓	1. The *XBP1*-116C/C genotype, a protective factor for bipolar disorder and schizophrenia, is decreased in patients with SCZ.	NO
Cheng et al., 2014	Asia	The *XBP1*-116C/G polymorphism was analyzed in different databases for case-control studies up to July 31, 2014.	*XBP1*-116C/G ↑	The *XBP1*-116C/G polymorphism is associated with an increased risk of psychiatric illness including bipolar disorder in the Asian population.	NM
Chen et al., 2004	China	The *XBP1*-197C/G polymorphism in unrelated patients (374 cases and 371 controls) was genotyped by PCR.	*XBP1*-197C/G ↑	1. *XBP1*-197C/G was significantly associated with SCZ, and the GG genotype frequency was higher than controls. 2. The G allele was significantly higher in the SCZ male groups; there were no significant changes in female groups.	NO
Kim et al., 2008	Korea	Five SNPs of *HSP70* in patients with SCZ (294 cases and 288 controls) were genotyped by using pyrosequencing method.	*HSP70-* rs2075799*G/A ↑	1. The rs2075799*G/A genotype was more represented in SCZ. 2. The T-A haplotype of rs2227956 and rs2075799 was significantly associated with SCZ.	NM
Kowalczyk et al., 2014	Poland	Polymorphisms of the *HSP-1* and *HSP-2* genes were genotyped by PCR-RFLP in drug-naïve patients with SCZ (203 cases and 243 controls) to examine the differences in genotype and allele frequencies.	*HSP-1* +190CC ↑	1. The *HSP-1*+190CC genotype and +190C allele were more represented in patients with SCZ. 2. Female patients with the *HSP-1*+190CC genotype have a higher risk of developing paranoid schizophrenia than male patients.	NO
Pae et al., 2005	Korea	Polymorphisms of the *HSP-1* and *HSP-2* were genotyped by PCR-RFLP in inpatients (161cases and 165 controls) to examine the differences in genotype and allele frequencies.	*HSP-2* ↑	1. *HSP-2* polymorphism contributed to the development of schizophrenia in a gene dose–dependent manner. 2. The *HSP-2* polymorphism conferred some susceptibility to schizophrenia in the Korean population.	YES

BiP, binding immunoglobulin protein; DLPFC, dorsolateral prefrontal cortex; GRP78, glucose regulatory protein 78; HSP, heat shock protein; PCR, polymerase chain reaction; PCR-RFLP, polymerase chain reaction-restriction fragment length polymorphism; p-JNK2, phosphorylated-c-Jun N-terminal kinase 2; RT-qPCR, real-time quantitative polymerase chain reaction; SCZ, schizophrenia; sXBP1, spliced X-box binding protein 1; UPR, unfolded protein response; WB, western blotting; XBP1, X-box binding protein 1; NM, not mentioned.

### A possible role of hypothalamic endoplasmic reticulum stress in schizophrenia-associated metabolic disorder?

**FIGURE 3:**
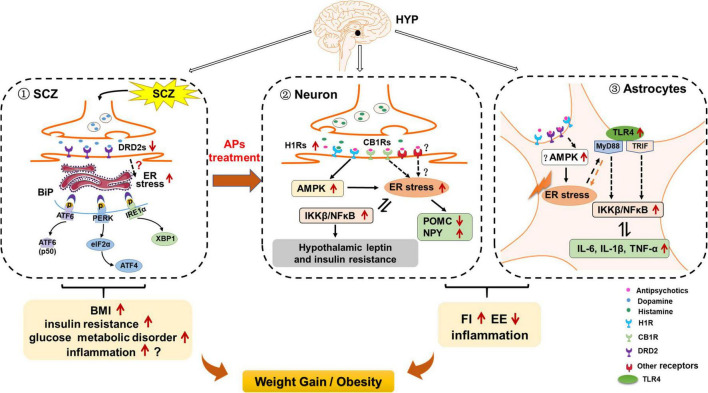
Possible role of hypothalamic endoplasmic reticulum (ER) stress in schizophrenia (SCZ)- and antipsychotic-induced weight gain/obesity. **(1)** Hypothalamic ER stress may be activated by decreased dopamine receptor D2 (DRD2) signaling, and these effects may be related to SCZ-related upregulation of body mass index (BMI), insulin resistance, and other metabolic disorders. Activated ER stress may also induce neuroinflammation; these effects may also be related to SCZ and its associated metabolic disorders. **(2)** In hypothalamic neurons, ER stress may contribute to antipsychotic-induced weight gain by inducing hyperphagia, decreasing energy expenditure, and inducing inflammation. Antipsychotics such as olanzapine and clozapine may activate hypothalamic ER stress by affecting H1 receptors (H1R) and CB1 receptors (CB1R), and activating adenosine 5′-monophosphate (AMP)-activated protein kinase (AMPK). On the one hand, activation of ER stress may inhibit proopiomelanocortin (POMC) processing, attenuate leptin signaling, and increase neuropeptide Y (NPY) and agouti-related protein (AgRP) expression, leading to increased food intake; reduced brown adipose tissue (BAT) thermogenesis and white adipose tissue (WAT) browning; increased inflammation; and thus weight gain/obesity Moreover, antipsychotic-induced ER stress activates inhibitor of nuclear factor kappa-B kinase subunit beta (IKKβ)/nuclear factor κB (NF-κB) signaling, leading to insulin and leptin resistance, and increased expression of pro-inflammatory cytokines such as interleukin 6 (IL-6), IL-1β, and tumor necrosis factor alpha (TNF-α). These effects may also contribute to antipsychotic-induced weight gain. **(3)** In hypothalamic astrocytes, antipsychotics such as olanzapine may induce ER stress possibly by acting on H1R, DRD2, and AMPK signaling. These effects may cause hyperphagia and decreased energy expenditure, resulting in weight gain. Antipsychotic-induced ER stress also leads to the increased TLR4 expression and activation of myeloid differentiation primary response 88 (MyD88)-independent/dependent signaling, which could stimulate the downstream IKKβ-NF-κB signaling activation and further causing the increased expression of pro-inflammatory cytokines such as TNF-α, IL-6, and IL-1β. These effects may be important for antipsychotic-induced obesity development.

Hypothalamic DRD2 signaling plays an essential role in the regulation of food intake, body weight, and glucose metabolism (Yonemochi et al., 2019; Ikeda et al., 2020). Several studies have proved that a decline in central dopaminergic activity significantly influences metabolic parameters such as BMI, glucose, and lipid metabolism in humans (Brunerova et al., 2013). Treatment with a DRD2 antagonist (L-741) but not a DRD3 antagonist could increase food intake and body weight of mice during anorexia (Klenotich et al., 2015). Mice with insulin receptors knocked out in the brain show reduced dopamine signaling, leading to behavioral disturbances (Kleinridders et al., 2015). Mice with *Drd2* gene knocked out exhibit impaired insulin secretion and glucose intolerance (García-Tornadú et al., 2010). Moreover, it has been proved that ER stress is involved in the dopaminergic neuronal function. In the substantia nigra pars compacta (SNpc) of *Drd2*-knockout mice, increased phospho-eIF2α was observed, showing that DRD2 inhibition may activate ER stress eIF2α signaling (Tinsley et al., 2009). Researchers reported that the DRD2 antagonist haloperidol increases PERK expression in a hepatocyte cell model (Lauressergues et al., 2012). On the contrary, activating DRD2 by levodopa attenuated α-syn-induced increased ER stress markers including ATF4, C/EBP homologous protein expression (CHOP), GRP78/BiP and XBP1 in SH-SY5Y neuronal cells (Song et al., 2017). These evidences suggested that reduced DRD2 signaling induced ER stress and these effects could be inhibited by DRD2 activation. Due to the importance of ER stress in regulating food intake, body weight and insulin secretion (Kim et al., 2012; Ajoolabady et al., 2022), it is suggested that in SCZ, reduced DRD2 signaling may lead to induction of ER stress, and these effects may largely contribute to SCZ-associated metabolic disorders such as increased BMI and glucose metabolic disorders (Figure 3).

Although there are controversial results, a meta-analysis suggests that SCZ is associated with the propensity to produce pro-inflammatory cytokines such as TNF-α, IL-6, and IL-1β in the brain (Momtazmanesh et al., 2019). Evidence has shown that DRD2 pathway regulates inflammation, and this effect cause metabolic disorders such as weight gain (Leite and Ribeiro, 2020). *Drd*2 knockout in mice produces a remarkable inflammatory response in the CNS (Shao et al., 2013). The DRD2 antagonist haloperidol increases TNF-α, IL-1β, and IL-6 expression *via* activating NF-κB signaling in SH-SY5Y cells exposed to hypoglycemia and hypoxia (Yang et al., 2022). Quinpirole, a DRD2 agonist, inhibits TLR4/NF-κB signaling and suppresses the expression of pro-inflammatory cytokines including TNF-α, IL-6, and IL-1β in the brain of an allergic rhinitis mouse model (Liu et al., 2021). ER stress *via* the IRE1-XBP1 and PERK pathways promotes inflammatory NF-κB signaling and increases TNF-α, IL-6, and IL-1β expression (Chipurupalli et al., 2021). Therefore, it is also possible that in SCZ, reduced DRD2 signaling activates ER stress, and these effects could activate inflammatory response and regulate body weight. Additional studies are necessary to evaluate this eventuality.

### Hypothalamic neuronal endoplasmic reticulum stress and antipsychotic-induced weight gain

Studies have revealed that treatment with antipsychotic drugs including olanzapine, haloperidol, clozapine, and aripiprazole increases the expression of IRE1 and PERK in human and mouse hepatocytes (Lauressergues et al., 2012; Weston-Green et al., 2018; Forno et al., 2020). Olanzapine or risperidone treatment causes significant ER stress in the pancreatic β-cell line of hamsters (Ozasa et al., 2013). This evidence suggests that antipsychotics activate ER stress. Our previous study showed that in human neuroblastoma SH-SY5Y cells, olanzapine treatment induces ER stress and activation of IKKβ-NF-κB signaling and secretion of the pro-inflammatory cytokines including TNF-α, IL-6, and IL-1β (He et al., 2019). Hence, olanzapine could directly act on neurons to induce ER stress and inflammation (He et al., 2019). The fact that the ER stress inhibitor 4-PBA suppresses olanzapine-induced ER stress and inflammation in SH-SY5Y cells suggests that olanzapine-induced inflammation is at least partly regulated by ER stress (He et al., 2019). In rats, both acute (1 day) and short-term (8 days) olanzapine treatment induce ER stress *via* PERK-eIF2α signaling; activate inflammatory IKKβ-NF-κB signaling; and augment TNF-α, IL-6, and IL-1β expression in the hypothalamus (He et al., 2019). These results suggest that olanzapine-induced ER stress and inflammation occur before rats are obese and could be a significant contributor rather than a consequence of obesity. Moreover, co-treatment with 4-PBA reduces olanzapine-induced hyperphagia and weight gain and inhibits olanzapine-induced ER stress and inflammation (He et al., 2019; Table 1). These findings indicate that olanzapine treatment activates hypothalamic neuronal ER stress and its related inflammatory IKKβ-NF-κB signaling, and these effects could lead to weight gain partly by increasing food intake. Hypothalamic ER stress also plays an important role in modulating energy expenditure. Numerous animal studies have revealed that obesogenic antipsychotics including olanzapine, clozapine, and risperidone reduce energy expenditure by inhibiting BAT thermogenesis, reducing tail artery vasoconstriction, and decreasing oxygen consumption and locomotor activity, all of which promote weight gain (Stefanidis et al., 2009; Bahr et al., 2015; Blessing et al., 2017). A study in humans reported that chronic olanzapine, risperidone, and quetiapine treatment (1, 3, 6, and 12 months) promotes weight gain with a hypometabolic state (Cuerda et al., 2011). Therefore, a reduction in energy expenditure induced by antipsychotics might be related to activation of hypothalamic ER stress. This eventuality should be evaluated in future studies. For example, investigating whether central inhibition of ER stress could reverse the antipsychotic-induced reduction in BAT thermogenesis and suppress the hypometabolic state in rodents would help to understand the role of ER stress in antipsychotic-induced decreased energy expenditure.

### Hypothalamic astrocyte endoplasmic reticulum stress and antipsychotic-induced weight gain

We recently found that olanzapine treatment also causes ER stress in cultured astrocytes, and induces activation of astrocytes in the rat hypothalamus (He et al., 2021). Astrocyte activation during olanzapine treatment occurs before weight gain onset, which indicates that astrocytic ER stress may be a contributor to antipsychotic-induced obesity (He et al., 2021). Moreover, we found upregulated expression of p-NF-κB, p-p38 (a MAPK), TLR4, MyD88, and p-ERK1/2 in olanzapine-treated cultured astrocytes. In rats, both short- and long-term olanzapine treatment significantly increase food intake and weight gain, accompanied by activated astrocytes and TLR4 signaling in the hypothalamus. In a pair-fed experiment, we found that olanzapine-treated rats do not exhibit significantly increased weight gain because of their limited access to food. However, the pair-fed olanzapine rats still exhibit activated astrocytes and TLR4 signaling in the hypothalamus, demonstrating that these changes are primarily caused by olanzapine rather than the secondary effects of hyperphagia or weight gain. Moreover, 4-PBA co-treatment inhibits olanzapine-induced hyperphagia, weight gain, astrocyte activation, and TLR4 signaling in the hypothalamus (He et al., 2021). These findings suggest that weight gain induced by olanzapine treatment may also be related to the hypothalamic astrocytes and TLR4 signaling, and these effects are mediated by the hypothalamic ER stress. After olanzapine activates ER stress, the TLR4 signaling pathway may be activated and thereby induce hyperphagia and weight gain (Figure 2). It is important to investigate whether astrocytic TLR4 knockout affects the hyperphagic and obesogenic effects of olanzapine in rodents.

The effects of other antipsychotics on astrocytes, ER stress, and TLR4 signaling, as well as their role in antipsychotic-induced obesity, are controversial. It has been reported that both acute (120 min) and short-term (7 days) treatment of clozapine, quetiapine, and brexpiprazole induce astroglial L-glutamate release and connexin 43 expression, suggesting that most second-generation antipsychotics affect astrocyte activity (Fukuyama and Okada, 2021). Clozapine treatment increases intracellular Ca^2+^ concentrations and decreases Ca^2+^ reentry in cultured cortical astrocytes and C6 cells (Kanda et al., 2016). Quetiapine treatment upregulates adenosine triphosphate (ATP) synthesis in astrocytes (Wang et al., 2014). Studies have reported that quetiapine inhibits astrocyte activation in APP/PS1 mice (Zhu et al., 2014) and in streptozotocin-induced diabetic mice (Wang et al., 2019). A previous study reported that chronic exposure to olanzapine (17–27 month period) in macaques significantly reduces the number of astrocytes in parietal gray matter by 20.5% (Dorph-Petersen et al., 2005). Moreover, a study in C6 astroglial cells reported that haloperidol increases extracellular levels of TNF-α and IL-1β and decreases IL-10, while risperidone decreases the release of TNF-α and IL-1β (Bobermin et al., 2018). This evidence suggests that antipsychotic drugs induce variable effects depending on the brain region, treatment period, animal models, and cell lines. Future studies should investigate the effect of antipsychotics such as olanzapine, risperidone and quetiapine on astrocytic ER stress in the hypothalamus and its role in antipsychotic-induced weight gain.

As previously mentioned, microglia also mediate inflammation and obesity development, and there is evidence that antipsychotics interact with microglia. Olanzapine and haloperidol activate microglia in the rat brain (Cotel et al., 2015). Risperidone inhibits microglial activation induced by IFN-γ (Racki et al., 2021). Clozapine reduces microglial activation induced by LPS in neuron-glia cultures (Hu et al., 2012). Clozapine, risperidone, and haloperidol do not affect cytokine expression levels in the absence of external stimuli to microglia (Giridharan et al., 2020). After induction of inflammation in microglia by poly (I:C), clozapine, risperidone, and haloperidol decrease the expression of IL-1α and IL-1β, and risperidone and haloperidol (but not clozapine) increase the expression of IL-6, IL-10, and TNF-α (Giridharan et al., 2020). Microglial ER stress and TLR4 signaling play a crucial role in the development of inflammation and obesity (Masson et al., 2015; Reis et al., 2015). Modulation of ER stress regulates the production of pro-inflammatory cytokines in microglia (Wang et al., 2017). Activation of TLR4 signaling by LPS induced a pro-inflammatory response in microglia (Zusso et al., 2019). TLR4 neutralization inhibited hypoxia-induced secretion of TNF-α and IL-1β in primary cultured microglia (Yao et al., 2013). However, the effects of antipsychotics on microglial ER stress and TLR4 signaling are currently unknown.

## Potential mechanisms in schizophrenia- and antipsychotic-induced endoplasmic reticulum stress during obesity development

As discussed above, hypothalamic ER stress may be activated by reduced DRD2 signaling that is part of the pathogenesis of SCZ and SCZ-related metabolic disorders, but additional studies are needed. The mechanisms by which reduced DRD2 signaling leads to ER stress activation are unknown. It has been suggested that in SCZ, DRD2 controls Ca^2+^ upregulation or release in neurons (Frégeau et al., 2013). Ca^2+^ is an important inducer of cellular ER stress (Krebs et al., 2015). Hence, in SCZ, DRD2 might regulate ER stress by affecting Ca^2+^. Studies that investigate alterations in hypothalamic ER stress markers and the Ca^2+^ level, DRD2 signaling in SCZ, and whether these effects could be inhibited by ER stress inhibitors are warranted. Moreover, we have reported that antipsychotics such as olanzapine activate hypothalamic ER stress in neurons (He et al., 2019) and astrocytes (He et al., 2021). Antipsychotics largely affect the energy sensor, AMPK, and have affinity for different neurotransmitter receptors such as DRD2 and H1R, which are expressed on both neurons and astrocytes and are related to ER stress. Could antipsychotic-induced ER stress be related to AMPK or these neurotransmitter receptors? In this section, we discuss the mechanisms by which antipsychotics induce ER stress.

### The possible mechanisms of antipsychotic-induced endoplasmic reticulum stress in neurons

Growing evidence suggests that AMPK may play a role in antipsychotic-induced ER stress. A previous study showed that ceramide-induced reduction in ER stress is regulated by AMPK in the VMH, but the exact mechanism is unclear (Martinez-Sanchez et al., 2017). Inactivation of AMPK within the VMH reduces ER stress, whereas constitutive activation of AMPK prevents triiodothyronine (T3)-induced inhibition of ER stress (Liu et al., 2020). These results suggest that AMPK could act as an upstream regulator of ER stress in the VMH (Liu et al., 2020). Previous studies have revealed that obesogenic antipsychotics such as olanzapine, clozapine, risperidone, and quetiapine activate hypothalamic AMPK and cause weight gain (Kim et al., 2007; He et al., 2014), but there are currently no data demonstrating that antipsychotics trigger hypothalamic ER stress *via* AMPK signaling. Furthermore, it is unclear which receptors antipsychotics affect to induce ER stress. Antipsychotics including olanzapine, clozapine, and quetiapine induce AMPK activation by blocking H1R (Kim et al., 2007). A previous study reported that H1R antagonism induces ER stress in MCF-7 cells (Jakhar et al., 2016). Therefore, antipsychotic-induced ER stress may be partly related to antagonism of hypothalamic H1R (Figure 1). More studies should investigate whether H1R activation could counteract antipsychotic-induced ER stress. Moreover, it has been reported that ACEA, a CB1R agonist, attenuates ER stress and inflammation in Neuro-2a neuroblastoma cells (Vrechi et al., 2018). Olanzapine treatment significantly reduces CB1R expression in the rat hypothalamus (Weston-Green et al., 2012). These findings indicate that ER stress may also be activated by antipsychotics such as olanzapine *via* CB1R pathways. Activation of hypothalamic ER stress leads to decreased POMC processing and disrupts leptin signaling. Previous studies have reported that olanzapine reduced the expression of POMC and leptin receptor as well as increased the expression of NPY and AgRP in the hypothalamus of rats (He et al., 2014; Zhang et al., 2014). Risperidone upregulated the mRNA expression of H1R, NPY, and AgRP in the hypothalamus (Lian et al., 2015). Hence, olanzapine and risperidone may activate ER stress by affecting H1R, CB1R, and AMPK. These effects may result in decreased POMC expression, increased NPY and AgRP expression and disrupted leptin signaling, therefore caused hyperphagia, decreased energy expenditure, weight gain, and inflammation (Figure 2). Furthermore, activation of IKKβ-NF-κB signaling through ER stress in the hypothalamus is associated with dysfunction of SOCS3 signaling (Zhang et al., 2008), which means central leptin and insulin resistance. Previous studies have suggested that antipsychotic treatment is associated with leptin resistance and insulin resistance at least partly by activating on H1R and DRD2 (Kim et al., 2007; Hahn et al., 2011); however, the molecular mechanisms are unknown. Antipsychotic-induced leptin and insulin resistance may be related to elevated hypothalamic ER stress. It is well known that leptin and insulin resistance are important contributors to hyperphagia, thermogenesis dysfunction and inflammation. Therefore, antipsychotic-induced ER stress may also cause leptin and insulin resistance, and these effects may worsen obesity and inflammation development during antipsychotic treatment (Figure 2).

### The potential mechanisms of antipsychotic-induced endoplasmic reticulum stress in astrocytes

The mechanisms by which antipsychotics induce inflammation, including secretion of pro-inflammatory factors and activation of TLR4 signaling in astrocytes, are not fully understood. Previous studies have reported that AMPK subunits such as α2, β2, and β1 are expressed in astrocytes (Turnley et al., 1999). Metformin reduces high-glucose-induced ER stress and inflammation partly by inhibiting AMPK signaling in primary cultured rat astrocytes (Wang et al., 2021). These results suggest that in astrocytes, AMPK may act upstream of ER stress. Therefore, it is possible that in astrocytes, olanzapine-induced ER stress may be related to AMPK during the development of inflammation and obesity (Figure 3). Future studies should explore whether inhibition of astrocytic AMPK suppresses antipsychotic-induced ER stress. Moreover, the receptors involved in antipsychotic-induced ER stress in astrocytes are unknown. As described above, in the hypothalamus, antipsychotics (e.g., olanzapine, clozapine, and quetiapine) induce AMPK activation by antagonizing H1R (Kim et al., 2007). A previous study reported that H1R is expressed in astrocytes (Xu et al., 2018). Histamine inhibits the secretion of pro-inflammatory factors including TNF-α and IL-1β, and these effects are completely abolished by co-treatment with the H1R antagonist cetirizine in primary cultured astrocytes (Xu et al., 2018). Hence, antipsychotic-induced activation of the inflammatory pathways and ER stress may be largely associated with the antagonism of astrocytic H1R.

Previous studies have proved that DRD2 inhibition is related to astrocytic inflammation. In LPS-stimulated astrocytes, bromocriptine, a DRD2 agonist, suppresses the expression of caspase-1, IL-1β, and TNF-α, changes that suggest DRD2 activation could reduce astrocytic inflammation (Tanaka et al., 2011; Zhu et al., 2018). In a Parkinson’s disease mouse model, the DRD2 agonist LY171555 specifically inhibits astrocytic inflammation by inducing the interaction of β-arrestin 2 and nod-like receptor protein 3 (NLRP3) (Zhu et al., 2018). In contrast, there are marked inflammatory responses in different brain regions of *Drd2*-deficient mice, including the hippocampus, spinal cord, striatum, and ventral midbrain (Shao et al., 2013). Ablation of *Drd2* in astrocytes activates astrocytes in the mouse substantia nigra and increased the mRNA expression of pro-inflammatory factors including IL-1β, IL-6, and cyclooxygenase 2 (COX-2) (Shao et al., 2013). Based on these findings, astrocytic DRD2 might be involved in astrocytic inflammation. Furthermore, in the SNpc of *Drd2*-knockout mice, there is significantly increased p-eIF2α, suggesting that DRD2 inhibition could activate ER stress (Tinsley et al., 2009). On the contrary, in SH-SY5Y cells, the DRD2 agonist bromocriptine reduces the expression of BiP, indicating that ER stress could be inhibited by DRD2 activation (Henderson et al., 2021). Given that olanzapine, haloperidol, risperidone, and ziprasidone block DRD2, antipsychotics may induce ER stress in astrocytes by antagonizing DRD2, resulting in activation of TLR4 and IKKβ-NF-κB signaling, and secretion of pro-inflammatory markers such as IL-6 and IL-1β, ultimately leading to inflammation and weight gain. Moreover, H1R, CB1R, and DRD2 are expressed on microglia (Dong et al., 2014; Huck et al., 2015; De Meij et al., 2021). H1R, CB1R, and DRD2 signaling in microglia mediates the expression of pro-inflammatory cytokines (Dong et al., 2014; Huck et al., 2015; De Meij et al., 2021), although the exact mechanisms are not clear. As has been mentioned, ER stress could work as a downstream of H1R and DRD2 signaling in astrocytes. However, in microglia, whether ER stress plays a role in H1R-, CB1R-, and DRD2-related inflammatory response is unknown. It is possible that antipsychotics affect microglial ER stress by antagonizing H1R, CB1R, and DRD2, therefore affecting the inflammatory response and body weight. Future studies should investigate whether inhibition of ER stress affects inflammatory cytokine expression induced by activation of H1R, CB1R, and DRD2 in microglia, and how ER stress inhibitors affect the effects of antipsychotics on microglial cytokine expression.

It is noteworthy that the reported effects of antipsychotics on inflammation are inconsistent and complex. In patients with SCZ, both stimulatory and inhibitory effects on inflammatory cytokines have been reported from studies on olanzapine, haloperidol, clozapine, and risperidone (Drzyzga et al., 2006). In rodents, clozapine increases the expression of IL-1β in rat serum (Sernoskie et al., 2022). Olanzapine induces the expression of IL-1β, IL-6, and TNF-α in the rat hypothalamus (He et al., 2019). The role of other antipsychotics on hypothalamic inflammation has not been elucidated fully. In astrocytes, olanzapine, risperidone, quetiapine, and haloperidol increase IL-1β (He et al., 2022a). In microglia, as has been reviewed, haloperidol and risperidone reduce IL-1β induced by poly (I:C) treatment, but increase IL-6 and TNF-α (Giridharan et al., 2020). These findings suggest that the effects of antipsychotics on inflammatory markers vary by cell type. The reasons are likely complicated. For example, antipsychotics such as clozapine, olanzapine, and quetiapine have affinity for DRD2 and H1R, which regulate inflammatory pathways in astrocytes and microglia. However, these receptors differentially regulate inflammatory pathways in different cell types. H1R activation decreases TNF-α and IL-1β in astrocytes (Xu et al., 2018), whereas H1R activation increases TNF-α and IL-6 in microglia (Dong et al., 2014). Activation of DRD2 suppresses the upregulation of IL-1β induced by LPS + ATP in primary cultured astrocytes (Zhu et al., 2018), whereas activation of DRD2 increases nitrite production in microglia (Huck et al., 2015). ER stress signaling could be downstream of H1R and DRD2 (Jakhar et al., 2016; Song et al., 2017). Studies suggest that ER stress differentially mediates inflammation in astrocytes and microglia. Inhibition of ER stress in astrocytes suppresses NF-κB activation induced by LPS and IFN-γ (Yanguas-Casás et al., 2014). However, in microglia, activation of ER stress by tunicamycin suppresses LPS-induced increases in IL-1β, IL-6, and TNF-α, whereas 4-PBA suppresses ER stress and promotes LPS-induced inflammation (Wang et al., 2017). Another study reported that suppression of ER stress by oxytocin inhibits TNF-α and IL-6 in microglia (Inoue et al., 2019). Therefore, the exact role of ER stress in microglia in regulating inflammation requires further investigation. The available evidence suggests that antipsychotics have differential effects on microglial and astrocytic inflammation by affecting H1R and DRD2 and their associated ER stress pathways. This may be an important reason why antipsychotics have been reported to produce pro- and anti-inflammatory effects in different studies. The overall effect of antipsychotics on inflammation in the hypothalamus is a combination of effects on neurons, astrocytes, and microglia. We suggest that ER stress in hypothalamic neurons and astrocytes may be related to antipsychotic-induced inflammation and obesity. Additional studies are needed to unravel how different doses of antipsychotics for different periods of time affect the astrocytic and microglial ER stress-inflammation pathways in the hypothalamus, to understand the mechanisms by which antipsychotics shows pro- or anti-inflammatory effects during different conditions.

## Endoplasmic reticulum stress inhibitors as therapeutic alternatives in managing metabolic alterations associated with schizophrenia and antipsychotic treatment

Schizophrenia is accompanied by metabolic disorders such as weight gain/obesity, glucose metabolism disorder, and leptin and insulin resistance. Antipsychotics are the main cause of these side effects. As discussed above, activation of hypothalamic ER stress *via* reduced DRD2 signaling may contribute to SCZ-associated increased BMI and insulin signaling dysfunction. Olanzapine activates ER stress in both neurons and astrocytes in the hypothalamus, and these effects could be related to olanzapine-induced weight gain/obesity (He et al., 2019, 2021). Clozapine, haloperidol, and risperidone activate ER stress, and these effects may involve antipsychotic-induced metabolic side effects (Lauressergues et al., 2012). These findings suggest that ER stress inhibitors are potential effective interventions against SCZ and antipsychotic related metabolic disorders such as weight gain/obesity and insulin signaling dysfunction. Previous studies have demonstrated that inhibition of ER stress by TUDCA or 4-PBA significantly reduces food intake, weight gain, abnormal glucose metabolism, and insulin and leptin resistance in obese rodents (Basseri et al., 2009; Zhou et al., 2016). Of note, 4-PBA and TUDCA have a very good safety profile and are used medications for patients. TUDCA and PBA could improve β-cell function in humans and increase insulin sensitivity in obese women and men (Kars et al., 2010; Sarvani et al., 2017). In antipsychotic-induced obese rodents, 4-PBA reverses olanzapine-induced weight gain and inflammation by inhibiting hypothalamic ER stress (He et al., 2019). Metformin, which suppresses weight gain at least partly by inhibiting ER stress (Li X. et al., 2021), inhibits olanzapine-induced weight gain in patients (Wu et al., 2008) and rodents (Hu et al., 2014). TUDCA inhibits olanzapine-induced insulin secretion (stimulated by glucose) in pancreatic β cells (Grajales et al., 2022). Overall, the ER stress inhibitors may be useful therapeutic alternatives to manage metabolic alterations associated with SCZ and antipsychotic treatment. Future studies should investigate the effects of TUDCA or 4-PBA on clozapine-, risperidone-, and quetiapine-induced weight gain and other metabolic disorders.

As has been reviewed, ER stress may also play an important role in the pathology of SCZ. ER stress inhibitors including 4-PBA and TUDCA are neuroprotective and significantly ameliorate the cognitive disorders in various neuropsychiatric disorders such as Alzheimer’s disease (Cuadrado-Tejedor et al., 2011; Dionísio et al., 2015) and cerebral ischemic injury (Qi et al., 2004). Pioglitazone, an antidiabetic agent, is an ER stress inhibitor and has been found to reduce the negative symptoms in patients with SCZ (Iranpour et al., 2016). Morin, an ER stress inhibitor, inhibits the IRE1-sXBP-1 signaling pathway induced by tunicamycin (an ER stress inducer) (Mo et al., 2019) and attenuates LPS + ketamine-induced SCZ-like behaviors in mice (Ben-Azu et al., 2019). The ER stress inhibitor, 4-PBA, could be a potential therapy for SCZ (Patel et al., 2017). These findings suggest that, in addition to managing SCZ-related metabolic disorders, the ER stress inhibitors may also effective in treating SCZ.

## Conclusion

The role of ER stress in SCZ and antipsychotic-induced weight gain/obesity has not been explored extensively. Based on evidence from clinical and pre-clinical studies, ER stress may be an important underlying mechanism for SCZ and antipsychotic-induced weight gain/obesity. Patients with SCZ show a reduction in hypothalamic DRD2 signaling, which may lead to activation of ER stress, thereby causing weight gain and dysregulated glucose metabolism. Antipsychotic drugs, in particular olanzapine, induce hypothalamic ER stress, and this effect is associated with inflammation and weight gain. Antipsychotics seems to induce hypothalamic ER stress by (1) obstructing POMC processing, attenuating leptin signaling, and increasing the expression of NPY/AgRP, therefore resulting in hyperphagia; (2) decreasing WAT browning and BAT thermogenesis, which inhibit energy expenditure; and (3) activating the MyD88-independent and MyD88-dependent pathways in astrocytes, resulting in increased secretion of pro-inflammatory cytokines. Based on the published evidence, antipsychotic-induced ER stress and the resulting inflammation may be related to the antipsychotic antagonism of H1R and DRD2. Taken together, hypothalamic ER stress could be a valuable target for mitigating SCZ and antipsychotic-induced metabolic side effects such as weight gain/obesity.

## Author contributions

MH and RZ wrote the manuscript. RZ, JF, RL, YZ, and BL revised the manuscript and figures. MH, GG, and TS provided the funding. All authors have contributed to and have approved the final manuscript.
